# Identification of Biomarkers for Defense Response to *Plasmopara viticola* in a Resistant Grape Variety

**DOI:** 10.3389/fpls.2017.01524

**Published:** 2017-09-05

**Authors:** Giulia Chitarrini, Evelyn Soini, Samantha Riccadonna, Pietro Franceschi, Luca Zulini, Domenico Masuero, Antonella Vecchione, Marco Stefanini, Gabriele Di Gaspero, Fulvio Mattivi, Urska Vrhovsek

**Affiliations:** ^1^Food Quality and Nutrition Department, Fondazione Edmund Mach San Michele all’Adige, Italy; ^2^Department of Agricultural and Environmental Sciences, University of Udine Udine, Italy; ^3^Computational Biology Unit, Fondazione Edmund Mach San Michele all’Adige, Italy; ^4^Genomics and Biology of Fruit Crop Department, Fondazione Edmund Mach San Michele all’Adige, Italy; ^5^Istituto di Genomica Applicata Udine, Italy; ^6^Center Agriculture Food Environment, University of Trento Trento, Italy

**Keywords:** biomarkers, *Plasmopara viticola*, hybrid, plant pathogen, Bianca, grapevine, resistance, metabolomics

## Abstract

Downy mildew (*Plasmopara viticola*) is one of the most destructive diseases of the cultivated species *Vitis vinifera*. The use of resistant varieties, originally derived from backcrosses of North American *Vitis* spp., is a promising solution to reduce disease damage in the vineyards. To shed light on the type and the timing of pathogen-triggered resistance, this work aimed at discovering biomarkers for the defense response in the resistant variety Bianca, using leaf discs after inoculation with a suspension of *P. viticola*. We investigated primary and secondary metabolism at 12, 24, 48, and 96 h post-inoculation (hpi). We used methods of identification and quantification for lipids (LC-MS/MS), phenols (LC-MS/MS), primary compounds (GC-MS), and semi-quantification for volatile compounds (GC-MS). We were able to identify and quantify or semi-quantify 176 metabolites, among which 53 were modulated in response to pathogen infection. The earliest changes occurred in primary metabolism at 24–48 hpi and involved lipid compounds, specifically unsaturated fatty acid and ceramide; amino acids, in particular proline; and some acids and sugars. At 48 hpi, we also found changes in volatile compounds and accumulation of benzaldehyde, a promoter of salicylic acid-mediated defense. Secondary metabolism was strongly induced only at later stages. The classes of compounds that increased at 96 hpi included phenylpropanoids, flavonols, stilbenes, and stilbenoids. Among stilbenoids we found an accumulation of ampelopsin H + vaticanol C, pallidol, ampelopsin D + quadrangularin A, *Z*-miyabenol C, and α-viniferin in inoculated samples. Some of these compounds are known as phytoalexins, while others are novel biomarkers for the defense response in Bianca. This work highlighted some important aspects of the host response to *P. viticola* in a commercial variety under controlled conditions, providing biomarkers for a better understanding of the mechanism of plant defense and a potential application in field studies of resistant varieties.

## Introduction

Downy mildew is one of the most destructive diseases of the grapevine caused by the biotrophic oomycete *Plasmopara viticola* (Berk. and Curt.) Berl. & de Toni. This pathogen was introduced from North America into Europe in the second half of the nineteenth century. The cultivated species *Vitis vinifera* is susceptible to *P. viticola*. Disease management strategies rely on the use of fungicides potentially harmful to humans and the environment (Negatu et al., 2016; Christen et al., 2017; Rortais et al., 2017). In some situations, chemical protection is also economically challenging, due to the costs of synthetic fungicides and the labor involved in spraying.

The pathogen is able to infect green tissues and establish biotrophism widely across the *Vitis* genus. Unlike the European *V. vinifera*, some accessions in North American wild species have evolved host resistance. Resistant accessions are able to activate defense responses upon pathogen infection, which culminate in localized necrosis, resulting into lower rates of sporangia release compared to susceptible accessions (Bellin et al., 2009; Polesani et al., 2010).

Resistant accessions of wild species have been crossed with cultivated varieties to introgress resistance. The use of resistant varieties is a promising strategy for viticulture to cope with downy mildew (Bisson et al., 2002). Among these, the variety Bianca is widely cultivated in Hungary, Moldova, and Russia and is one of the few resistant accessions in which the genetic basis of resistance has been elucidated (Bellin et al., 2009). Bianca is an hybrid between Bouvier and the resistant grapevine Villard Blanc. It was obtained in 1963 (Csizmazia and Bereznai, 1968), and officially registered for use in wine production in 1982 (Kozma and Dula, 2003). A large part of the resistance phenotype of Bianca is explained by the *Rpv3* locus, located in chromosome 18. In Bianca and in all known resistant descendants of the Villard Blanc the *Rpv3* locus controls the ability to trigger a localized hypersensitive response (HR) soon after the initiation of the infection (Bellin et al., 2009; Di Gaspero et al., 2012). HR in the proximity of infection sites confines biotrophic pathogens, restricting their endophytic growth (Jones and Dangl, 2006). Early inducible responses include cell wall deposition, release of reactive oxygen species and hypersensitive cell death (HR) at the infection site, controlled by interactions between avirulence gene products and plant receptors, and it can be the result of multiple signaling pathways (Heath, 2000).

Plant defense responses require energy and activation of signaling molecules, primarily supplied by primary metabolism of carbohydrates, organic acids, amines, amino acids, and lipids (Bolton, 2009; Rojas et al., 2014). HR also stimulates the expression of defense responses near the infected area and the onset of systemic acquired resistance (Greenberg and Yao, 2004). Several studies have shown the importance of secondary metabolites for expressing plant defense, often related to specific functions such as toxicity against pathogens, or acting as signal molecules after stress (Bennett and Wallsgrove, 1994; Gershenzon and Dudareva, 2007). The induction of stress-related metabolites known as phytoalexins contributed to the inhibition of biotrophic pathogens in resistant grapevines (Dercks and Creasy, 1989; Derckel et al., 1999; Slaughter et al., 2008; Godard et al., 2009; Ferri et al., 2011; Gessler et al., 2011). Stilbenes is a class of phytoalexins that provided active compounds with antifungal activity against various pathogens, including *P. viticola* (Dercks and Creasy, 1989). The pattern of stilbene accumulation upon *P. viticola* infection differs among *Vitis* species. Stilbene concentration showed earlier and higher increase in resistant varieties as compared to susceptible ones. In other cases, downy mildew resistance was observed in the absence of stilbene accumulation (Keller, 2015). This suggests the necessity to investigate which secondary metabolites play a key role in resistance and which of them are reliable biomarkers of the defense response in resistant varieties.

We expect that several classes of primary and secondary metabolites are modulated in Bianca during the defense response to *P. viticola*. In this scenario, metabolomics is the most suitable approach for monitoring a wide range of molecules. Indeed, several metabolomics studies have been already reported in grapevine. Some of them aimed at highlighting intervarietal variation in berry composition (Mulas et al., 2011; Gika et al., 2012; Degu et al., 2014; Teixeira et al., 2014; Bavaresco et al., 2016). Other studies aimed at identifying metabolite changes in infected leaves (Batovska et al., 2009; Ali et al., 2012; Becker et al., 2013; Algarra Alarcon et al., 2015). However, the metabolite changes that are brought about by the resistance mechanism have not yet been fully described.

In this work, we monitored metabolite changes in leaf discs of the resistant variety Bianca after infection with a suspension of *P. viticola*, with the aim of discovering biomarkers for specific stages of the host defense. In particular, we evaluated both primary and secondary metabolism at 12, 24, 48, and 96 h post-inoculation (hpi). We used existing protocols of LC-MS/MS for identification and quantification of lipids and phenols, and GC-MS for identification and semi-quantification of volatile organic compounds (VOCs). Moreover, we validated a new GC-MS protocol for the identification and quantification of primary compounds, including organic acids, amino acids, amines, sugars, and lipids, which yielded 48 metabolites in Bianca leaf discs.

## Materials and Methods

### Plant Material

Metabolite analyses were performed using leaves from two-node cuttings of the cultivar Bianca. The mother plants were held at Fondazione Edmund Mach grape collection, San Michele all’Adige, Italy (46°12′0″N, 11°8′0″E). Own-rooted vines (*n* = 45) were grown in potted soil in controlled greenhouse conditions. Water was supplied by drip irrigation in order to avoid premature infections of downy mildew on leaves. At the stage of 12-leaf shoots, the plants were sorted into three homogenous groups; each group represented a biological replicate. At the time of the experiment plants were healthy, with no evidence of foliar diseases.

### Artificial Inoculation of Leaf Discs and Incubation under Controlled Conditions

The third, fourth, and fifth fully expanded leaves beneath the apex were detached from each plant, rinsed with ultrapure water. From each leaf, 1.1 cm diameter discs were excised with a cork borer and placed randomly onto wet paper in Petri dishes with the abaxial side up. Around 100 discs per condition per time point (i.e., 12 hpi, inoculated, biological replicate 1 = 100 discs) were used. Leaf discs were left to equilibrate at 21°C for 12 h after punching and prior to inoculation. *P. viticola* spores were collected from natural infected leaves in an untreated vineyard in 2014 and immediately frozen at -20°C. They were propagated by infecting a susceptible variety and collecting fresh sporulation. After sporulation, the fresh spores were immediately used to prepare the experiment suspension. Discs were sprayed with *P. viticola* inoculum suspension at 1 × 10^6^ sporangia/mL. Sealed Petri dishes were incubated in a growth chamber at 21°C until sampling. Mock inoculated control were prepared with ultrapure water. Leaf discs were sampled at 12, 24, 48, and 96 hpi/mock, then ground under liquid nitrogen to obtain a frozen powder. Three biological replicates were sampled at each time point.

### Targeted Primary Compound Analysis and Method Validation

#### Sample Preparation

The extraction of primary metabolites was carried out according to Fiehn et al. (2008) with some modifications. Briefly, 0.1 g of fresh leaf powder was subjected to extraction by adding 1 mL of cool (-20°C) extraction solvent, composed of isopropanol/acetonitrile/water (3:3:2 v/v/v). A 20 μL aliquot of a solution containing palmitic-D3, nicotinic-D4, and glucose-D7 (1000 mg/L) was added as an internal standard. The extraction mixture was vortexed for 10 s, shaken at 4°C for 5 min and centrifuged at 12,000 *g* for 2 min at 5°C. A second round of extraction was carried out following the same procedure. The two supernatants were merged and re-suspended in a final volume of 5 mL using the extraction solvent. A total of 250 μL of supernatant was placed in a 1.5 mL Eppendorf tube and evaporated to dryness under N_2_. The residue was re-suspended in 500 μL of acetonitrile/water (50:50 v/v), vortexed for 10 s, sonicated and centrifuged at 12,000 *g* for 2 min. The supernatant was then transferred into a 1.5 mL Eppendorf tube and dried out under N_2_. The dried extract was subject to derivatization, first by adding 20 μL of methoxamine hydrochloride in pyridine (20 mg/mL) to inhibit cyclization of reducing sugars and shaken at 30°C for 1 h; then by adding 80 μL of *N*-methyl-*N*-trimethylsilyl-trifluoroacetamide with 1% trimethylchlorosilane for trimethylsilylation of acidic protons and shaken at 37°C for 30 min. Finally, 5 μL of a solution containing decane and heptadecane (1000 mg/L) were added in order to monitor the chromatographic analysis and the instrumental conditions. The derivatized extract was then transferred into vials for analysis. One microliter of derivatized extract was injected for GC/MS analysis.

#### Instrumental Conditions

Analyses were performed using a Trace GC Ultra combined with a mass spectrometer TSQ Quantum GC and an autosampler Triplus (Thermo Electron Corporation, Waltham, MA, United States). A RXI-5-Sil MS w/Integra-Guard^®^(fused silica) (30 m × 0.25 mm × 0.25 μm) column was used for compound separation. Helium was used as the carrier gas at 1.2 mL/min and the injector split ratio was set to 1:10. The injector, transfer line and source temperature were set to 250°C. The initial oven temperature was kept at 65°C for 2 min, increased by 5.2°C/min to 270°C and held at 270°C for 4 min. These conditions were shown to represent a good compromise in order to obtain a not excessively long chromatographic run, a high number of compounds and good peak separation. The mass spectrometer was operated in electron ionization mode. Data acquisition was performed in full scan mode from 50 to 700 *m/z*. Data processing was performed using XCALIBUR^TM^ 2.2 SOFTWARE.

#### Method Validation

The method for primary metabolites was validated according to the currently accepted US Food and Drug Administration (FDA) bio-analytical method validation guide (US Department of Health and Human Services, 2001). Validation assays were established on calibration standards and quality control (QC) samples prepared as a pool of grape samples, extracted and derivatized according to the procedure described above. QC samples were used to evaluate the recovery of each compound and the stability of sample, intra- and inter-day variability, and to evaluate the efficiency of the extraction procedure. The standard mix was used to determine the limit of detection (LOD), limit of quantification (LOQ), and linearity range for each compound. Matrix calibration curves built using QC samples were compared with solvent calibration curves. Matrix effect (ME) values were determined using the slope ratios: ME% = 100 × (1 - slope solvent calibration curve/slope matrix calibration curve) (Kwon et al., 2012). LOQ and LOD were evaluated at the concentration in which the quantifier transition presented a signal-to-noise (S/N) ratio of >10 and >3, respectively. Intra- and inter-day variability were evaluated using the coefficient of variation (CV%) of QC samples injected 10 times on 1 day and then for 5 consecutive days. The recovery test was estimated on 10 spiked grape samples and calculated as the average of the “measured value/expected value” ratio (%). Each compound was identified and quantified against the standard, using one, or in the case of a few compounds, two specific *m/z* characteristics for the individual metabolite (extracted ion monitoring) and excluding saturated fragments. The fragments used for quantitation and the linear retention index (RI) are reported in Supplementary Table S1. Compounds were expressed as mg/kg of fresh leaves.

### Targeted Lipid Compound Analysis

Lipid analysis was carried out according to Della Corte et al. (2015), using Folch’s extraction method (Folch et al., 1957; Della Corte et al., 2015) with some modifications. Briefly, 0.3 mL of methanol were added to 0.1 g of fresh leaf powder and vortexed for 30 s, then 0.6 mL of chloroform containing butylated hydroxyl toluene (500 mg/L) were added, followed by the addition of 10 μL of internal standard (docosahexaenoic acid 100 μg/mL). Samples were placed in an orbital shaker for 60 min. After the addition of 0.25 mL of water, samples were centrifuged at 3600 rpm for 10 min. The total lower lipid-rich layer was collected and re-extracted by adding 0.4 mL of chloroform/methanol/water 86:14:1 v/v/v. The samples were centrifuged at 3600 rpm for 10 min, the total lower lipid-rich layer was collected. Both chloroform fractions were merged and evaporated to dryness under N_2_. Samples were re-suspended in 300 μL of acetonitrile/2-propanol/water (65:30:5 v/v/v) containing the internal standard cholesterol at a concentration of 1 μg/mL and transferred into a HPLC vial. Separation was performed using a UHPLC Dionex 3000 (Thermo Fisher Scientific, Germany), with a RP Ascentis Express column (15 cm × 2.1 mm; 2.7 μm C18) purchased from Sigma, following a 30 min multistep linear gradient following Della Corte et al. (2015). The UHPLC system was coupled with an API 5500 triple-quadrupole mass spectrometer (Applied Biosystems/MDS Sciex) equipped with an electrospray ionization (ESI) source. Compounds were identified using Analyst Software based on their true reference standard, retention time and qualifier and quantifier ion, and were quantified using their calibration curves and expressed as mg/kg of fresh leaves.

### Targeted Phenolic Compound Analysis

Phenolic compounds were determined according to Vrhovsek et al. (2012), with some modifications. Briefly, 0.4 mL of chloroform and 0.6 mL of methanol:water (2:1) were added to 0.1 g of fresh leaf powder. A 20 μL aliquot of gentisic acid (50 mg/L) and rosmarinic acid (50 mg/L) were added as internal standards. The extraction mixture was shaken for 15 min in an orbital shaker, then centrifuged for 5 min at 15,000 *g* at 4°C. The upper aqueous-methanolic phase was collected. The extraction was repeated by adding 0.6 mL of methanol and water (2:1 v/v) and 0.2 mL of chloroform; the samples were centrifuged for 5 min at 15,000 *g* at 4°C. The aqueous-methanolic phase was collected and combined with the previous one. Both fractions were merged and evaporated to dryness under N_2_. Samples were re-suspended in 500 μL of methanol and water (1:1 v/v), centrifuged and transferred carefully into an HPLC vial. Chromatographic analysis was performed using a Waters Acquity UPLC system (Milford) with a Waters Acquity HSS T3 column (100 mm × 2.1 mm; 1.8 μm) following Vrhovsek et al. (2012). Mass spectrometry detection was performed on a Waters Xevo triple-quadrupole mass spectrometer detector (Milford) with an electrospray ionization (ESI) source (Vrhovsek et al., 2012). Compounds were identified based on their reference standard, retention time and qualifier and quantifier ion, and were quantified using their calibration curves and expressed as mg/kg of fresh leaves. Data processing was performed using Waters MassLynx V4.1 software.

### Volatile Compound Analysis

Volatile compounds were extracted with solid phase microextraction, using a method adapted from Matarese et al. (2014) and Salvagnin et al. (2016). The extraction was carried out with some modifications; briefly, 0.1 g of fresh leaves were placed in 10 mL glass vials with 2 mL of buffer (0.1 m Na_2_HPO_4_ and 50 mM citric acid; pH 5), 0.2 g of NaCl, and 5 μL of 1-heptanol (25 mg/L) as internal standard. Samples were kept at 60°C for 20 min and compounds in the headspace were captured for 35 min at 60°C. A Trace GC Ultra gas chromatograph coupled to a Quantum XLS mass spectrometer (Thermo Electron Corporation, Waltham, MA, United States) was used to separate the compounds with a fused silica Stabilwax^®^-DA column (30 m × 0.25 mm i.d. × 0.25 μm) (Restek Corporation, Bellefonte, United States). The headspace was sampled using 2-cm DVB/CAR/PDMS 50/30 μm fiber from Supelco (Bellefonte, PA, United States). The compounds were desorbed in the GC inlet at 250°C for 4 min. The GC oven parameters were set following Salvagnin et al. (2016). The MS detector was operated in scan mode (mass range 40–450 *m/z*) with a 0.2 s scan time and the transfer line to the MS system was maintained at 250°C. Data processing was performed using XCALIBUR^TM^ 2.2 SOFTWARE. For the identification of volatile compounds we used letter “A” for compounds with comparable mass spectra and retention time to those of the pure standard, “B” for those with a RI match on a similar phase column with the database NIST MS Search 2.0, and “C” for those identified in the mass spectral database NIST MS Search 2.0 (Sumner et al., 2007). The experimental linear temperature RI of each compound was calculated using a series of *n*-alkanes (C10-C30) in the same experimental conditions as the samples. The results were expressed in a semi-quantitative manner and expressed in μg/kg using 1-heptanol as the internal standard.

### Data Analysis

Statistical analysis and data visualization were performed with custom R scripts (R Core Team, 2017). Missing values were imputed with a random value between zero and LOQ. The concentrations were transformed using the base 10 logarithm, in order to make data distribution more normal-like (van den Berg et al., 2006). Principal component analysis (PCA) was performed on the obtained multidimensional dataset, after mean centering and unit scaling, using the FactoMineR and Factoextra R packages (Lê et al., 2008; Kassambara and Mundt, 2017). The *t*-statistic was computed using the Stats package (R Core Team, 2017), while network visualization exploited the ggraph package (Pedersen, 2017).

## Results

In leaf discs inoculated with *P. viticola* and in mock-inoculated controls, we identified 176 compounds (Supplementary Table S2) belonging to acids (18), amino acids (13), amines and others (3), sugars (14), carnitines (1), sterols (3), fatty acids (14), glycerolipids (4), glycerophospholipids (4), sphingolipids (1) prenols (1), benzoic acid derivates (4), coumarins (2), phenylpropanoids (6), dihydrochalcones (1), flavones (1), flavan-3-ols (9), flavonols (11), stilbenes and stilbenoids (14), and other phenolics (2). All these metabolites were annotated with identification level 1 (with standards) and their concentration was expressed as mg/kg of fresh leaves. The volatile acids (3), alcohols (7), aldehydes (9), benzenoids (4), ketones (4), terpenoids (14), other VOCs (5), and unknown VOCs (5) were semi-quantified as the equivalent of the internal standard (1-heptanol) and their concentration was expressed as μg/kg of fresh leaves (Supplementary Table S2). The concentration reported represents the average value of three biological replicates ± standard error. For the identification of VOCs, we reported the confidence levels for metabolite identification defined by the Metabolomics Standards Initiative (Sumner et al., 2007): level A is assigned to compound for which the mass spectrum and the retention time match with the one of the pure standard; level B indicates that the RI of the compound and of the reference standard matches on a similar phase column; level C is assigned when the compounds mass spectrum is available into mass spectra databases (Supplementary Table S2).

### Validation Results of the Primary Compound Method

Unlike for lipid, phenolic, and volatile compounds, a validated protocol for identification and quantification of primary metabolites in grapevine leaves was missing at the beginning of this study. We thus adopted a method established by Fiehn et al. (2008) on grape berries and performed a validation step to confirm the identity of each compound in a leaf matrix. All the standards were injected to obtain their fragmentation patterns and to calculate their retention indices. The calculated retention indices and mass spectra were compared with the NIST MS Search 2.0 database. The method was validated with the injection of relative standards for 96 compounds: 29 acids, 17 amino acids, 12 amines and others, 24 sugars and 14 fatty acids (Supplementary Figure S1). All the validation results are summarized in Supplementary Table S1. The ME values evaluated by comparing the calibration curves (matrix and solvent) were in the range between -20 and 20%, except for salicylic acid, citric acid, glycine, beta-alanine, tyrosine, fructose, and *myo*-inositol, which slightly exceeded the limit of ±20% established by the validation method guide; this value can be considered as insignificant, because it is close to the relative standard deviation values of repeatability (European Commission, 2011). Intra- and inter-day repeatability were evaluated for each compound and expressed as CV%. The value should not exceed 15% for intra-day and 20% for inter-day; again in this case we had very good results, except for oxalic acid (intra-day 18.2%; inter-day 26.4) and malonic acid (intra-day 15.2%; inter-day 44.6%). The recovery ranges were over 90% for 74 compounds, between 80 and 90% for 13 compounds, between 70 and 80% for four compounds, and between 50 and 70% for five compounds. Using solvent calibration curves we evaluated the linearity ranges and the LOD and LOQ limits for each compound reported in Supplementary Table S1. In general, we obtained good validation results for the method, which make us confident about the possibility of applying the method for accurate quantification of primary compounds in different matrices.

The fatty acid derivatization step can modify the profile, with the formation of oxidation or isomerization products (Rigano et al., 2016) and as previously reported, the best option is to use trimethylsilyl diazomethane, with the production of methyl esters (FAMEs), avoiding the poor separation of fatty acid compounds and substantial interference (Topolewska et al., 2015). In our method, we validated all the compounds following the derivatization used by Fiehn et al. (2008), but we found a consistent residue in blank injections of some compounds, such as palmitic acid, stearic acid, and arachidic acid in particular during the sample runs. Due to this interference, we were not able to correctly quantify fatty acid compounds in our matrix using the GC-MS method therefore their quantification was performed using LC-MS/MS.

### Metabolite Changes during the Defense Response

**FIGURE 1:**
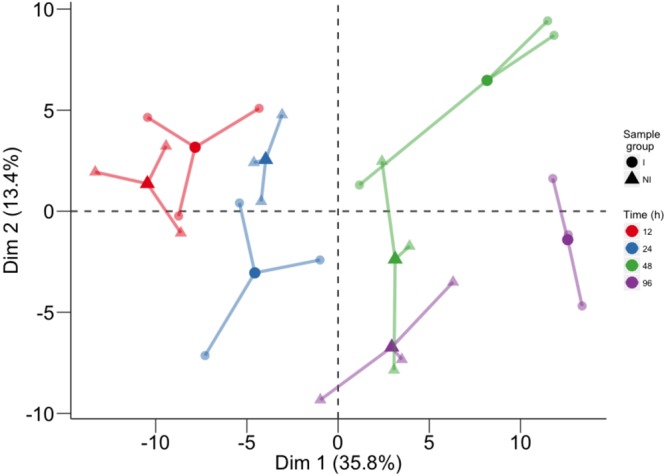
Principal component analysis performed on the log10 of the concentration of all analyzed compounds. For each time point, three biological replicates (smaller dots) are represented for each condition (circle: inoculated samples; triangle: not inoculated) and linked with their means (larger dots). Each time point is represented with a different color: red for samples collected at 12 hpi, blue for 24 hpi samples, green for 48 hpi samples, and violet for 96 hpi samples.

Global metabolite changes in the resistant host upon pathogen inoculation were first visualized by using PCA (**Figure 1**). In the plot, the position of the three biological replicates and their average is reported for each time point (12, 24, 48, and 96 h) for inoculated and not inoculated leaves. PCA of all compounds revealed good separation between the factors of the study and the temporal evolution was clearly captured by the first PCA component (Dim 1), accounting for 35.8% of total variance. The second component (Dim 2), accounting for 13.4% of total variance, discriminated leaf discs undergoing a defense response to *P. viticola* from mock inoculated controls (**Figure 1**).

**FIGURE 2:**
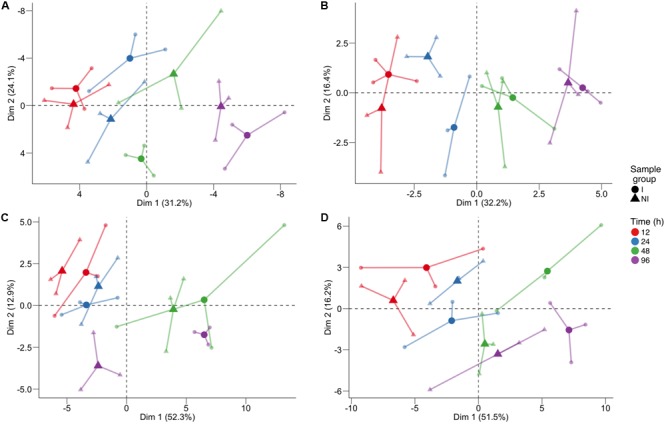
Principal component analysis of the log10-transformed metabolite concentration of individual classes: **(A)** primary compounds, **(B)** lipids, **(C)** phenol compounds, and **(D)** volatile compounds. The analysis was performed on the log10 of the concentration. For each time point, three biological replicates (smaller dots) are represented for each condition (circle: inoculated samples; triangle: not inoculated) and are linked with their means (larger dots). Each time point is represented with a different color: red for samples collected 12 h post-infection (hpi), blue for 24 hpi samples, green for 48 hpi samples, and violet for 96 hpi samples.

In order to identify which class of metabolites was responsible for this separation, we performed PCA (**Figure 2**) separately for primary metabolites (**Figure 2A**), lipids (**Figure 2B**), phenolic compounds (**Figure 2C**), and VOCs (**Figure 2D**). Again the time trend was clearly distinguishable (captured by the first component), and also a good separation between the two conditions can be noticed for specific time points for the different classes of compounds. Indeed, we observed for primary compounds a clear separation between the two conditions along the second dimension (which captured 24.1% of the total variance) at 48 hpi and, looking at the two components (explaining a total of 55.3% of the variance), possibly at 24 hpi (**Figure 2A**). Lipids showed the greatest differences at 24 hpi, where the inoculated and control samples are separated mainly along the second component, explaining 16.4% of the total variance (**Figure 2B**). Phenols were involved in the plant response only later, at 96 hpi, with the first component capturing 52.3% of the variance and possibly explaining both the time course and the differences between the two conditions (**Figure 2C**). Finally, PCA of VOCs separated inoculated and not inoculated samples at 48 hpi, and at 96 hpi, mainly along the first component, which explains 51.5% of the variance due to both the time trend and the differences between the two conditions in the last two time points (**Figure 2D**).

**FIGURE 3:**
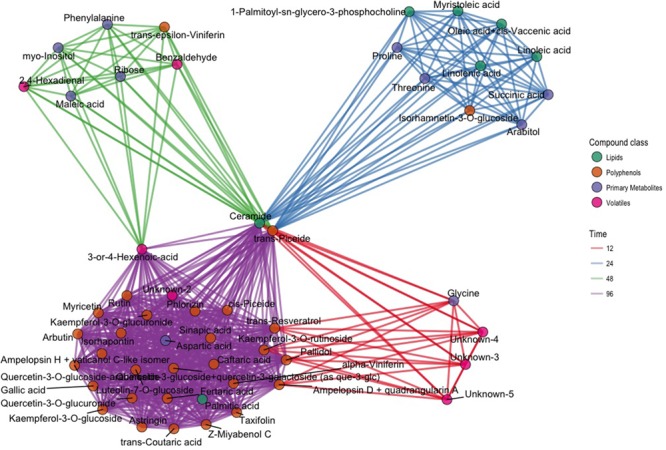
Network plot representing the 64 most different metabolites in inoculated and not inoculated samples, corresponding to compounds having an absolute value of the *t*-statistic greater than 3 (|*t*| > 3). Each of the 64 compounds is represented with a dot; dots belonging to different classes are in different colors (green: lipids; brown: polyphenols; violet: primary compounds; pink: volatile compounds). Metabolites with differences after infection modulated at the same time point are linked using the color link of the specific time point (red: 12 hpi; blue: 24 hpi; green: 48 hpi; violet: 96 hpi).

In order to select biomarkers for each specific stage of the defense response, we computed the *t*-statistic for all the metabolites for each time point, since it takes into account both the difference between the means and the estimate of the biological variability. To concentrate on the compounds most different between inoculated and control samples, we focused on a subset of metabolites (176) and time points (4) having an arbitrary absolute value for the *t*-statistic greater than 3 (|*t*| > 3): 64 values of the *t*-statistic satisfied our constraint. In terms of compounds we identified 53 metabolites, which were different between inoculated and control samples in at least one time point (Supplementary Figure S2 and Table S3). The results of this analysis are represented in the network of **Figure 3**. The network contains 53 nodes, each one representing one metabolite. A link is drawn between two metabolites only if both metabolites have |*t*| > 3 at the same time point. In the same visualization the class of the compound is highlighted by the color of the node and the time course information by the color of the link (**Figure 3**). Time point specific cliques are characterizing the structure of the network: the metabolites shown in one of these cliques show differences between the two conditions at that specific time point. The smaller number of nodes in the network at 12 hpi indicates that metabolic changes were minimal at this time point, only involving a small group of volatile compounds, glycine, ampelopsin D + quadrangularin A, *trans*-resveratrol, kaempferol-3-*O*-rutinoside, and pallidol. Several lipids and primary metabolites were highly modulated at 24 hpi, as already shown in **Figure 2**. No lipids were highly modulated at 48 hpi, and many polyphenols were highly modulated at 96 hpi. Moreover, it is apparent that ceramide and *trans*-piceid show a central position in the network, meaning that these compounds were highly modulated at all the time points. Some interesting results are represented by metabolites connected with two different colored links; these were different at two time points, as exemplified by *trans*-resveratrol (**Figure 3**).

To further investigate this interesting subset of compounds and as further check for both the selection criterion and the visualization proposed, we explored the trends of log10 concentration over time for some key metabolites. These plots show results consistent with the network representation (Supplementary Figure S3). Ceramide concentration in inoculated leaves was already higher at 12 hpi compared with controls and reached the highest concentration at 96 hpi (0.32 mg/kg; Supplementary Table S2). *Trans*-piceid concentration was already high at 12 hpi and reached the highest concentration at 48 hpi (5.29 mg/kg) (Supplementary Table S2). Among the polyphenols, *trans*-𝜀-viniferin was the compound that was modulated earliest at 48 hpi, while other trimeric and tetrameric stilbenoids, such as ampelopsin H + vaticanol C, pallidol, ampelopsin D + quadrangularin A, *Z*-miyabenol C and α-viniferin for example, were modulated at 96 hpi (Supplementary Figure S3). Accumulation of *trans*-resveratrol occurred early after infection (12 hpi), and was followed by a decrease in concentration at 24 and 48 hpi, and a resumption of accumulation at 96 hpi (Supplementary Figure S3).

A considerable number of compounds belonging to each class increased in concentration over time in both inoculated and not inoculated samples (Supplementary Table S2); this would explain the high variance in the first dimension of PCA, which is associated with the time course (**Figure 1**). The progressive accumulation of stress-related compounds in leaf discs, regardless pathogen inoculation, can be explained by other stresses affecting the tissues as a consequence of leaf removal, punching of the leaf lamina, and artificial conditions of leaf disc incubation. We found accumulation of some lipid compounds, such as arachidic acid, oleanolic acid, and uvaol, in inoculated and control samples (Supplementary Table S2). In polyphenols we also observed an accumulation of flavonols and some trimers and tetramers belonging to the stilbene and stilbenoid class during the first 48 h, irrespective of pathogen infection, and then differentiation at 96 hpi (Supplementary Table S2). These results are consistent with previous reports of metabolite changes caused by mechanical wounding (Chitarrini et al., 2017).

## Discussion

Grapevine and *P. viticola* interaction is still poorly understood in terms of metabolites: there is the need to improve the knowledge about how the plant system is perturbed after stress. In this study, a metabolomic approach has revealed major changes in primary and secondary metabolism of a resistant grape variety during the defense response to *P. viticola*. The identification of biomarkers, specific of four stages of the defense response, from 12 to 96 hpi, reflected a progression of physiological events that bring about resistance.

In addition to the importance of secondary metabolites in the fight against pathogens, the role of primary metabolism needs to be taken into account, since it is not only an energy provider but also regulates defense responses in plants in the presence of potential pathogens or pathogen-derived elicitors (Rojas et al., 2014). We expected that a defense response against an endophytic biotroph could be triggered only after the establishment of intimate contact between pathogen haustoria and host plasma membranes. In fact, we observed minimal metabolite changes in the host within 12 hpi, compatibly with a scenario in which *P. viticola* oospores/zoospores take several hours to germinate on the leaf lamina, target the stomata, form appressoria, break through the cell wall of mesophyll cells and develop functional haustoria. We identified a few biomarkers for this very early stage of host–pathogen interaction. Most of them were volatile compounds, which may interfere with the pathogen endophytic invasion of mesophyll air spaces.

The classes of biomarkers specific to 24 and 48 hpi suggested that early host responses to *P. viticola* were being set in place during those stages. We detected a sharp shift in primary metabolism. Leaf discs undergoing the defense response showed a cumulative amount of sugars, organic acids, and amino acids 6.7% higher than controls at 12 hpi, 9.4% higher at 24 hpi, 14.1% higher at 48 dpi, and 11% lower at 96 dpi. These data suggest that most of the metabolic effort for containing pathogen infection was carried out by 48 hpi and the metabolic cost for this effort was paid at 96 hpi. In leaf discs undergoing the defense response, organic acids were 13.3% higher than controls at 12 hpi and 24.6% higher at 24 hpi. Vice versa, sugars and amino acids were consistently lower at the same time points in leaf discs undergoing the defense response (-2.3 and -12.1% sugars at 12 and 24 hpi, respectively; -8.9 and -19.9% amino acids at 12 and 24 hpi, respectively). At 48 hpi sugars and amino acids were 33.9 and 42.3% higher in leaf discs undergoing the defense response compared to controls. The cost of expressing defense has been shown in barley as a peak of respiration rate during the expression of host resistance to *Blumeria graminis* (Brown and Rant, 2013).

Primary metabolism is important for energy supply but it also has a role providing precursors of secondary metabolites, building blocks of PR proteins, and components of the defense signaling cascade (Rojas et al., 2014). Less et al. (2011) found different regulation of specific genes in *Arabidopsis* related to primary metabolism, due to abiotic and biotic stress response; in particular, up-regulation of genes involved in energy production processes and down-regulation of genes associated with assimilatory processes was found (Less et al., 2011). We found changes in primary compounds at 24 and 48 hpi, in particular we observed an interesting modulation for proline. In *Arabidopsis*, both supply and catabolism of proline are components of salicylic acid-mediated resistance, contributing to cell death in response to *Pseudomonas* (Deuschle et al., 2004; Cecchini et al., 2011). The role of proline in the Bianca grapevine variety after *P. viticola* infection should be elucidated with further experiments, however, for the moment we can identify this molecule as a putative biomarker.

Lipids represent a class of compounds with structural diversity and complexity. They are critical components of plant cell membranes and provide energy for metabolic activities. We found changes at 24 hpi in particular with a faster decrease in some unsaturated fatty acids after *P. viticola* infection. Ceramide started accumulating very early in infected samples compared to the control, and continued to accumulate after biotic stress up to 96 hpi; it was previously reported that ceramides can be essential as signaling molecules in the activation of defense-related plant programmed cell death (Kachroo and Kachroo, 2009; Berkey et al., 2012).

Subsequently, secondary metabolism was affected more strongly by the pathogen, with changes in the volatile compounds at 48–96 hpi and at the latest at 96 hpi in phenolic compounds. Some phenolic compounds, such as phenylpropanoids and flavonoids, have previously been identified and considered responsible for distinguishing the resistant cultivar Regent from the susceptible Trincadeira (Ali et al., 2012). We found higher concentrations of these compounds in our infected samples compared with the control at 96 hpi (Supplementary Table S2); this result suggests their involvement as biomarkers of resistance to the pathogen in the Bianca grapevine. *Trans*-resveratrol production in grapevine leaves after pathogen infection was identified by Langcake and Pryce (1977). It has been demonstrated that *trans*-resveratrol is a precursor of fungal toxicity compounds identified as phytoalexins; these compounds can be produced by grapevine leaves after abiotic and biotic stress and can be used in the grapevine as a marker of resistance against pathogens (Jeandet et al., 2002). The accumulation of *trans*-resveratrol at 12 hpi in our infected samples can reflect the role of this molecule as a precursor of other toxic molecules, and the very early *trans*-resveratrol accumulation in Bianca is probably due to a rapid response to the pathogen. In our study we found a major increase in some molecules deriving from resveratrol, such as *trans*-𝜀-viniferin at 48 hpi and subsequently *trans*- and *cis*-piceid, isorhapontin, ampelopsin H + vaticanol C-like isomer, α-viniferin and pallidol at 96 hpi. During the first hpi, a low accumulation of viniferins (grapevine specific stress related metabolites) was found after pathogen infection, probably due to their accumulation at a later stage (4–7 days after inoculation), as previously described (Pezet et al., 2004; Jean-Denis et al., 2006; Slaughter et al., 2008). The time course of accumulation of these viniferins is in full agreement with several experiments reviewed by Bavaresco et al. (2012), beginning with the synthesis of resveratrol and progressing with the formation of dimers and then the higher oligomers. Such a path requires growth through the subsequent addition of one resveratrol unit to an existing dimer, leaving one part of the initial structure unchanged. The biosynthesis of dimers and higher oligomers appears to be important for resistance, in agreement with the observations of Malacarne et al. (2011) in a segregating population of Merzling × Teroldego. This paper indeed highlighted a negative correlation between the content of different oligomers and the percentage of sporulation upon infection, while this was not the case for the monomers *trans*-resveratrol and *trans*-piceid, which were also found in sensitive genotypes with high sporulation. Moreover, the importance of viniferin oligomers is further confirmed by the concentration values required to induce inhibition of mildew development recently reported by Gabaston et al. (2017).

In our study, the peak of accumulation of phenolic compounds at 96 hpi was anticipated at 48 h by the accumulation of phenylalanine in inoculated samples (**Figure 3** and Supplementary Table S2). Phenylalanine is the precursor of the phenylpropanoid pathway, leading to the synthesis of flavonoids and stilbenes by stilbenes, two classes of compounds that increased at 96 (Sparvoli et al., 1994; Flamini et al., 2013).

Among the volatile compounds, we found an increase in benzaldehyde production at 48 and 96 hpi in inoculated samples (Supplementary Figure S2) Benzaldehyde is considered as a growth suppressor and spore inhibitor, with activity against *Botrytis cinerea*, also at a low concentration (Martínez, 2012). Benzaldehyde also promotes salicylic acid accumulation, induces expression of PR proteins and increases TMV resistance in tobacco (Ribnicky et al., 1998). The higher concentration we found in infected Bianca samples at 48 and 96 hpi (around 1.5 times higher compared to the control) suggests its involvement as a putative biomarker against *P. viticola* growth or diffusion.

Based on our results, we can argue that all the compounds significantly differentiated in infected samples have a role in Bianca-*P. viticola* interaction. In particular, 53 metabolites have been identified as putative biomarkers in hybrid Bianca grapevine leaves after *P. viticola* infection. Some of them are known biomarkers of resistance (viniferins). Among the others, some are likely to be putative biomarkers of resistance in Bianca leaf discs after *P. viticola* infection, such as benzaldehyde and proline.

To the best of our knowledge, this is the first time that an extensive metabolomic study has been undertaken using a resistant grape variety to better understand metabolic perturbation after *P. viticola* infection, finding early stage biomarkers for different chemical classes of metabolites. These results can represent a starting point for better understanding grapevine resistance and can lead to discoveries regarding new mechanisms for plant–pathogen interaction between the grapevine and *P. viticola*.

We also obtained good results for method validation in relation to the identification and quantification of 97 primary compounds belonging to different chemical classes: acids, amino acids, amines, sugars, and fatty acids, using a GC-MS method for separation and identification. The method can easily be applied to further analysis for the identification and quantification of primary compounds in different matrices.

## Author Contributions

GC, LZ, AV, MS, GD, FM, and UV designed the experiment. GC, GD, LZ, and AV performed the experiment. GC, ES, and DM did the extractions, analytical analysis, and data treatment. GC, ES, and DM developed and validated the method. SR and PF conducted all the statistical analyses. All authors discussed the results and implications and commented on the manuscript at all stages.

## Conflict of Interest Statement

The authors declare that the research was conducted in the absence of any commercial or financial relationships that could be construed as a potential conflict of interest.
