# Supplementary material for: Identification of Biomarkers for Defense Response to Plasmopara viticola in a Resistant Grape Variety
Source: Front Plant Sci. 2017 Sep 5;8:1524. doi: 10.3389/fpls.2017.01524 (PMC5591819; doi:10.3389/fpls.2017.01524)
Supplement: Supplementary file 4 [file Image_1.PDF]

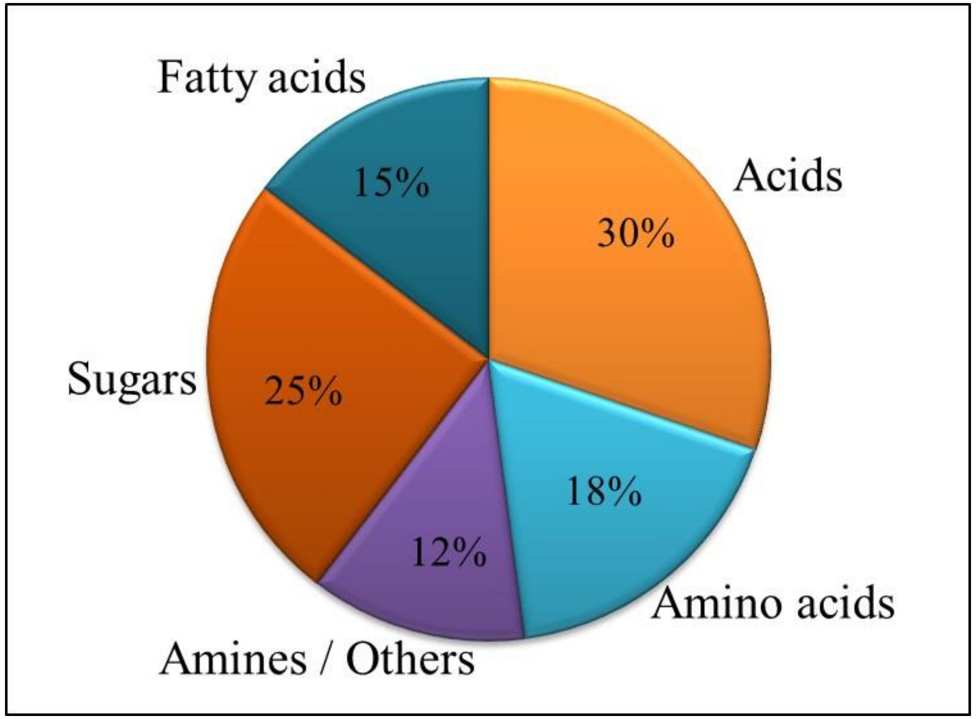

Fig. S1: Different classes of primary compounds taken into account for the GC-MS/MS validation method.
