# Supplementary material for: Identification of Biomarkers for Defense Response to Plasmopara viticola in a Resistant Grape Variety
Source: Front Plant Sci. 2017 Sep 5;8:1524. doi: 10.3389/fpls.2017.01524 (PMC5591819; doi:10.3389/fpls.2017.01524)
Supplement: Supplementary file 5 [file Image_2.PDF]

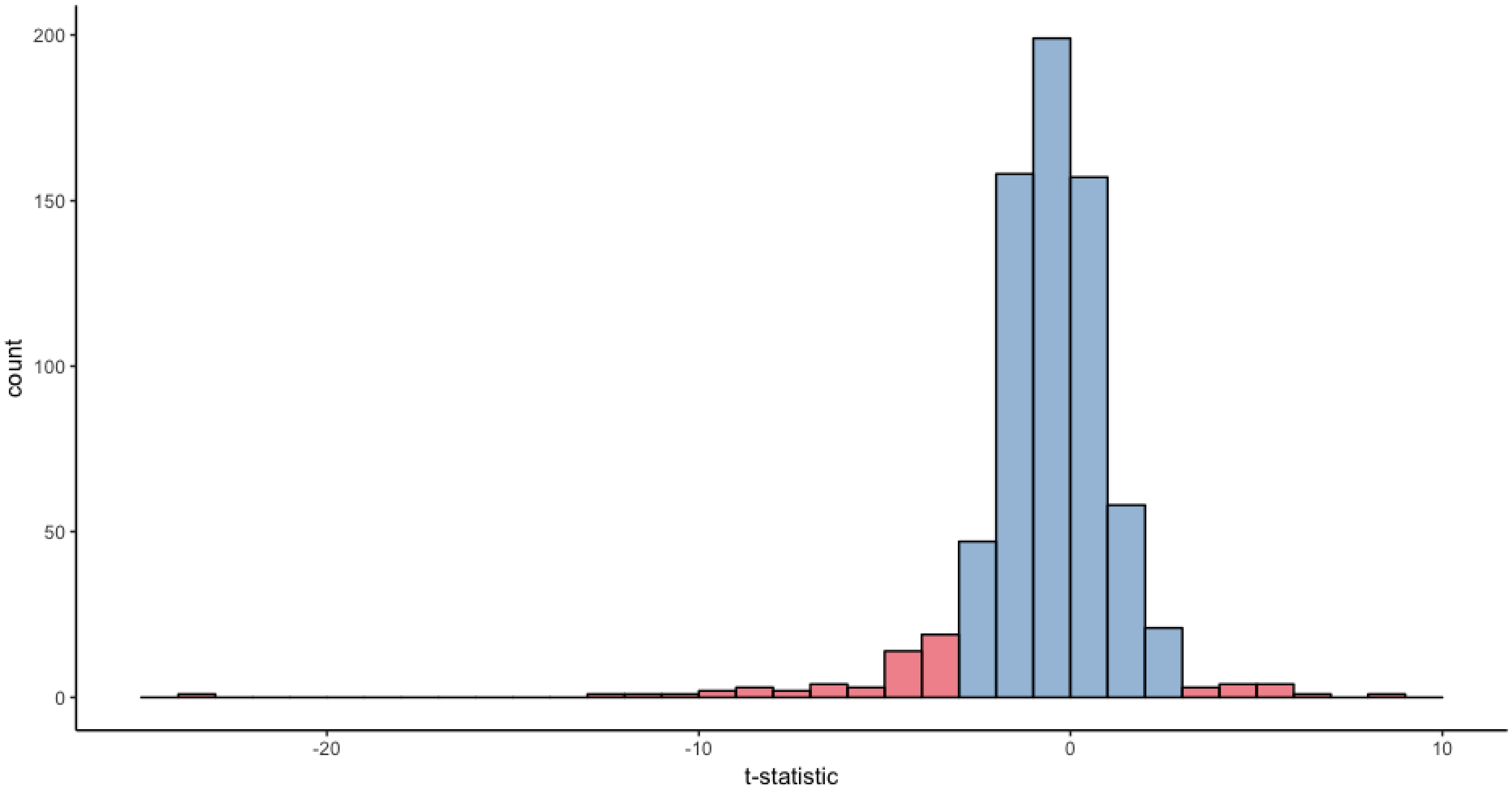

Fig. S2: t-statistic values of our metabolites. For network analysis we took into account only metabolites with |t|>3.
