# Supplementary material for: Identification of Biomarkers for Defense Response to Plasmopara viticola in a Resistant Grape Variety
Source: Front Plant Sci. 2017 Sep 5;8:1524. doi: 10.3389/fpls.2017.01524 (PMC5591819; doi:10.3389/fpls.2017.01524)
Supplement: Supplementary file 6 [file Image_3.PDF]

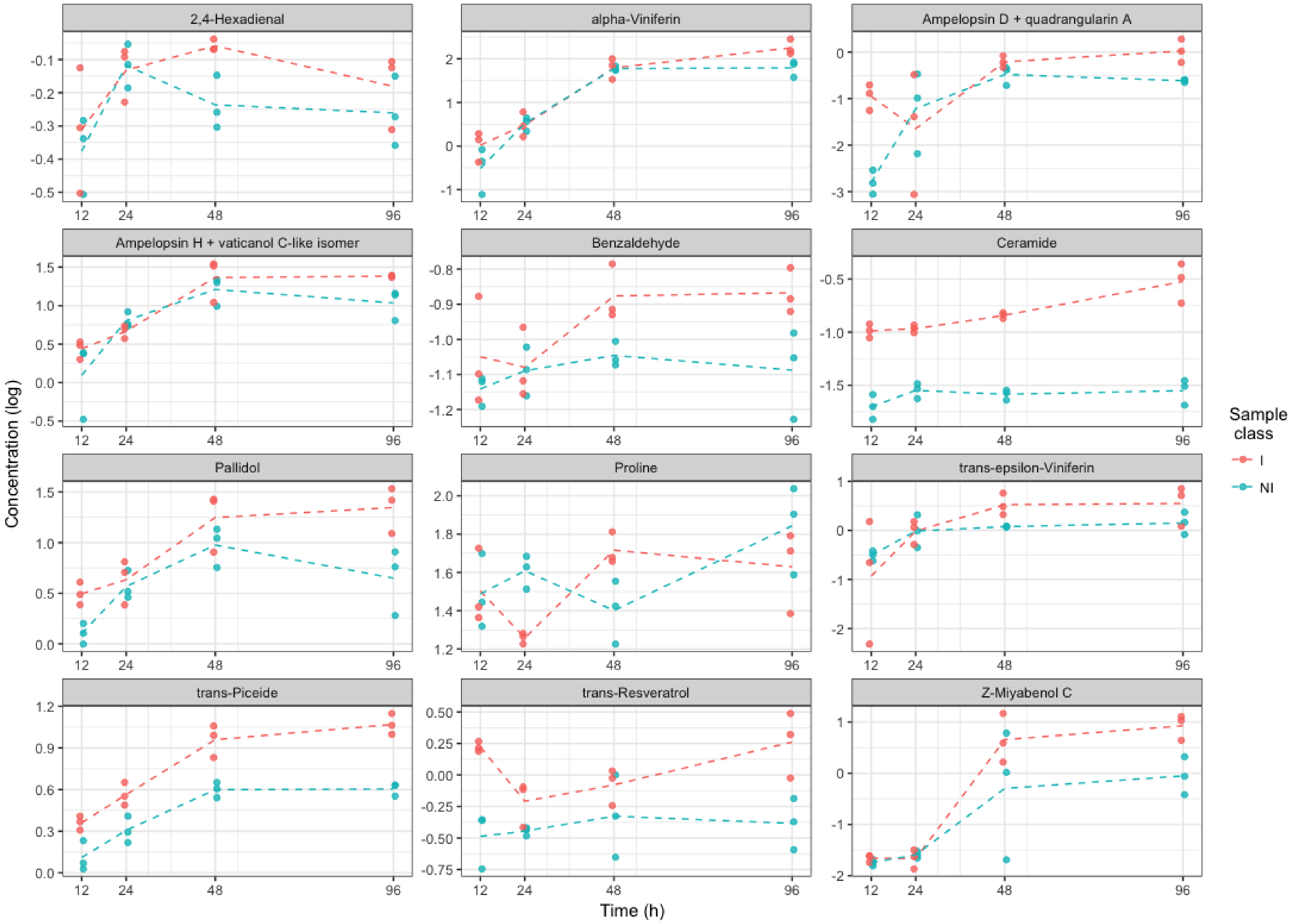

Fig. S3: Graphs for specific metabolites. The Log10-transformed metabolite concentration of the three biological replicates over time is represented for each of the metabolites (I= inoculated samples; NI= not inoculated samples). The line represents the mean of the three biological replicates. Where missing values were present they were imputed with a random value between zero and LOQ.
